# Focused liquid ultrasonography in dropsy protocol for quantitative assessment of subcutaneous edema

**DOI:** 10.1186/s13054-023-04403-y

**Published:** 2023-03-18

**Authors:** Weiqing Zhang, Yanting Gu, Yujin Zhao, Jun Lian, Qian Zeng, Xiaoting Wang, Jun Wu, Qiuying Gu, Chinese Critical Care Ultrasound Study Group (CCUSG)

**Affiliations:** 1 Department of Critical Care Medicine, Ruijin Hospital, Shanghai Jiao Tong University School of Medicine, No.197, Rui Jin Er Road, Shanghai, China; 2 Shanghai Jiao Tong University School of Nursing, Shanghai, China; 3 Department of Critical Care Medicine, Peking Union Medical College Hospital, Chinese Academy of Medical Sciences and Peking Union Medical College, Beijing, China; 4 Chinese Critical Care Ultrasound Study Group, Beijing, China

**Keywords:** Edema, Ultrasonography, Critically ill patients

## Abstract

**Background:**

Although subcutaneous edema is a common symptom of critically ill patients, it is still underreported due to the lack of a systematic method for evaluating it. The present study aims to describe the occurrence and distribution of subcutaneous edema, as well as the risk factors associated with it, in critically ill patients using the focused liquid ultrasonography in dropsy (FLUID) protocol, and to assess their impact on ICU mortality.

**Methods:**

The FLUID protocol and the pitting test were performed on general ICU patients in China. Cohen’s Kappa coefficient and Bland–Altman plots were used to evaluate the agreement between the two methods at each measurement site and between the whole-body subcutaneous edema scores, respectively, while a repeated measures ANOVA was performed to compare the differences between the two methods in whole-body and body-part measurements. A generalized linear model was used to evaluate the risk factors for subcutaneous edema development and the relationship between subcutaneous edema severity and ICU mortality.

**Results:**

A total of 145 critically ill patients were evaluated using both approaches, of whom 40 (27.6%) experienced subcutaneous edema. Over 1440 measurements, it was found that ultrasound discovered more subcutaneous edema than the pitting test (ultrasound: 522[36.3%], pitting test: 444[30.8%], *χ*^2^ = 9.477, *p* = 0.002). The FLUID protocol scored edema severity significantly higher than the pitting test in the whole body and specific body parts, including the abdominal wall, thighs, chest wall, and hands. Subcutaneous edema exhibited gravity-dependent distribution patterns, particularly in the abdominal wall. The APACHE II, NT-proBNP, serum creatinine, and sepsis were independent risk factors for subcutaneous edema development. The score of ultrasonic subcutaneous edema was related to ICU mortality.

**Conclusions:**

The FLUID protocol provides a comprehensive strategy for the semi-quantitative assessment of subcutaneous edema in critically ill patients. In detecting the onset and severity of edema, ultrasound was found to outperform the pitting test. Subcutaneous edema showed a gravity-dependent distribution pattern, and its severity was associated with mortality.

## Introduction

Critically ill patients have many risk factors for the development of subcutaneous edema, such as increased vascular permeability due to the inflammatory response, fluid resuscitation and fluid overload, cardiac insufficiency, and renal impairment [1–3]. Danziger et al. found that 18% of critically ill patients had subcutaneous edema when admitted to the intensive care unit (ICU), and 6% had both pulmonary and subcutaneous edema [4]. Patients with subcutaneous edema tended to be older, with a greater prevalence of heart failure, diabetes, acute respiratory distress syndrome, acute lung injury, sepsis, and acute kidney injury [5, 6].

Although it is common practice to examine hospitalized patients for subcutaneous edema, this aspect of the job has not received adequate attention (7). There is also a lack of studies on the subcutaneous edema state in critically ill patients. The main reason is that there is no internationally unified standard for the evaluation and measurement of subcutaneous edema in critically ill patients [7], and the existing methods are unsuitable for assessing the overall systemic edema state of critically ill patients. Nowadays, the pitting test (PT) represents the most extensive physical examination and grading method for subcutaneous edema [8]. The severity of edema, from slight to very marked, is traditionally reported on a four-point scale that reflects the amount of time for the skin to return to normal after applying pressure [9]. However, the grading of pitting edema is subjective [8]. The development of alternative objective assessment tools is required.

While ultrasound has been widely used in assessing pulmonary edema [10], its application in the evaluation of subcutaneous tissue edema, especially in critically ill patients, has rarely been reported. Ultrasound emits sound waves to create images of subcutaneous tissues. When the sound wave reaches the boundary of an acoustically different tissue, such as fluid or soft tissue, part of the energy is reflected back in a manner dependent on the acoustic difference [11]. This allows the exploration of subcutaneous structures and their changes by high-frequency ultrasound probes [12]. This led to investigations of the feasibility and reliability of ultrasonography for assessing subcutaneous edema in specific body parts [13–17]. However, data on its value in assessing the systemic edema state of critically ill patients are still limited. Therefore, the aims of the present study were: (1) to explore the strategy of systematic assessment of subcutaneous edema by ultrasound; (2) to observe the distribution characteristics of subcutaneous edema and identify factors associated with its occurrence; and (3) to evaluate its impact on ICU mortality.

## Methods

### Design, setting, and population

This prospective observational study was conducted in a general ICU in Shanghai, China, from October 2021 to June 2022. The study was approved by the Ruijin Hospital Ethics Committee (Reference No. 2020-147), and all patients provided written informed consent (either directly or through an appropriate surrogate). Patients aged ≥ 18 years with an anticipated ICU stay of at least 24 h were eligible for screening and inclusion. Patients were excluded if they did not have arms or legs for PT and ultrasound assessments or had wounds, fractures, lesions, burns, or bleeding at the measurement points. This study was registered in the Chinese Trial Registry (ID: ChiCTR2100051560) and was drafted in compliance with the STROBE guidelines [18].

### General demographic and clinical data

Information on the general demographic and clinical features of eligible patients was collected within 24 h of admission to the ICU and was documented in the electronic medical record system. This information included age, body mass index (BMI), ICU admission diagnosis, surgical status, surgical site, central venous pressure (CVP), N-terminal B-type natriuretic peptide (NT-proBNP), creatinine, prealbumin, total protein, albumin, the Acute Physiology and Chronic Health Evaluation II (APACHE II) Score, level of consciousness, mechanical ventilation, vasoactive drugs, and comorbidities (cardiac dysfunction, acute kidney injury, and intra-abdominal infection). The prognosis of the patients was recorded when they moved out of the ICU.

### “Focused liquid ultrasonography in dropsy” (FLUID) protocol

**Fig. 1:**
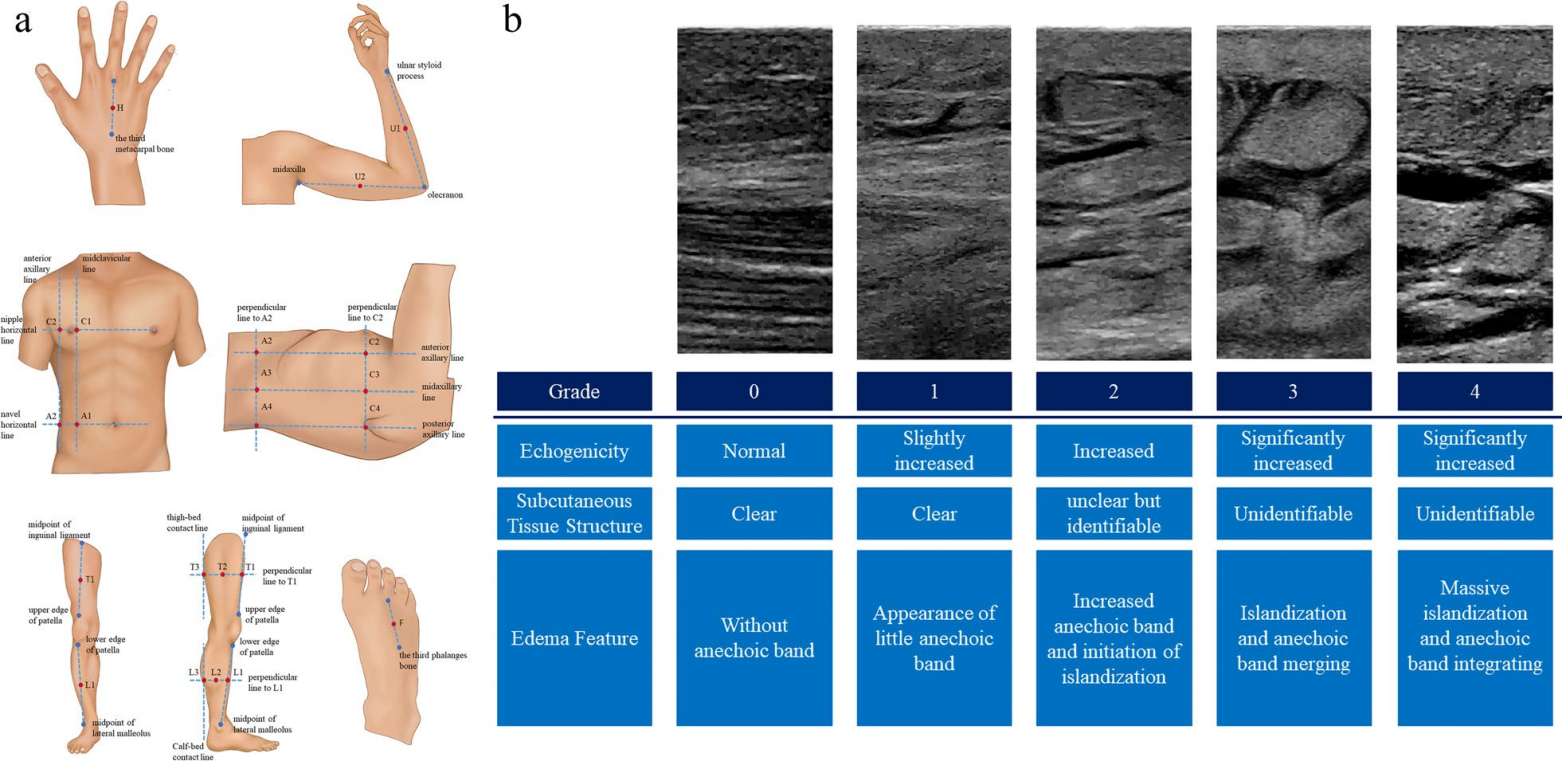
Illustration of two essential elements of FLUID protocol. The 36 measurement sites of the FLUID protocol (**a**); Illustrations of ultrasonic subcutaneous edema grade (**b**)

Subcutaneous tissue was scanned within 24 h after ICU admission using a Philips ultrasound machine (IU22, USA) with a 5–12 MHz linear transducer, with trained and qualified researchers performing the assessment. Before the procedure, the patients were positioned supine with their elbows, wrists, knees, and muscles relaxed, and their palms and toes facing or pointing to the ceiling. The 36 sites of assessment are shown in Fig. 1a.

A 5-point scale of ultrasonic subcutaneous edema grade (USEG) was utilized to assess subcutaneous edema at each site based on echo intensity, tissue transparency, and fluid properties (Fig. 1b). The transducer was placed parallel to the sagittal plane of the body except at sites H and F (Fig. 1a), where it was perpendicular. The performers used the "i-SCAN" button to automatically optimize gain and time gain compensation (TGC). The focus point was the center of the images, and grayscale compression was adjusted automatically and ranged from 60 to 70%. An ultrasonic subcutaneous edema score (USES) was obtained by adding scores from 36 subcutaneous sites (0–144). Each regional score was calculated by summing the site scores of the hands (0–8), arms (0–16), thoracic wall (0–32), abdominal wall (0–32), thigh (0–24), calves (0–24), and feet (0–8). The total regional score was then divided by the number of sites in each region to obtain the average score (0–4) for each body part. Higher values indicated worse subcutaneous edema. Prior to the formal examination, the reliability of FLUID score assessments between two operators was determined in 10 patients. The inter-rater and intra-rater correlation coefficients were 0.88 and 0.90, respectively.

### Pitting test

Following the ultrasound evaluation, researchers who were blinded to the ultrasound evaluation performed a PT on patients [9]. The PT was modified to improve its objectivity and reliability, and the measurement was repeated at the 36 anatomical sites used in the FLUID protocol. A pressure of 150 mmHg was applied for 5 s on a 1.5 cm-diameter probe using a Pull & Push gauge (Model: DS2-20N, ZHIQU Precision Instruments Co., Ltd., Dongguan, China) to maintain the pressure. The time taken for the skin to return to normal after the release of the pressure was scored as follows: Grade 0 = pitting returns to normal immediately; Grade 1 = pitting takes 30 s to return to normal; Grade 2 = 30 to 60 s; Grade 3 = 60 to 90 s; Grade 4 = 90 to 120 s [8]. Edema scores were calculated as in the FLUID protocol.

### Statistical analysis

Data were entered into SPSS version 19 (IBM Corp., Armonk, NY, USA) for analysis. The Shapiro–Wilk method was used to test the distribution normality of numerical variables. Values were presented as mean (standard deviation) (SD), while non-normally distributed values were presented as median ([interquartile range] [IQR]), percentage proportions as well as total numbers. The agreement among the extent of edema for each measurement site as determined by the PT and the USEG was evaluated using Cohen’s Kappa coefficient. A Kappa value < 0.40 indicates weak agreement, 0.40–0.75 moderate-good agreement, and > 0.75 excellent agreement [19]. A Bland–Altman plot was created to assess agreement between USEG and PT on the whole body. The repeated measures ANOVA was used to compare the differences between USEG and PT on the whole body and body parts. Chi-square tests were used to analyze the differences in the composition ratios of edema grades between USEG and PT. A generalized linear model was used to identify risk factors for subcutaneous edema occurrence and to test the performance of the USES in predicting ICU mortality, with the results reported as area under the receiver operating characteristic (AUC-ROC) plots. For all analyses, a two-sided P-value < 0.05 was considered significant.

### Sample size

Given the findings of the pretest of a USEG standard deviation of 10 and a correlation coefficient of 0.90 between the USEG and PT using repeated measures ANOVA analysis, for a two-sided *P*-value of 0.05 and 90% power, it was determined that the study would require a sample size of at least 40 to differentiate mean differences of 7 between the USEG and PT measures [20].

## Results

### Patients

**Fig. 2:**
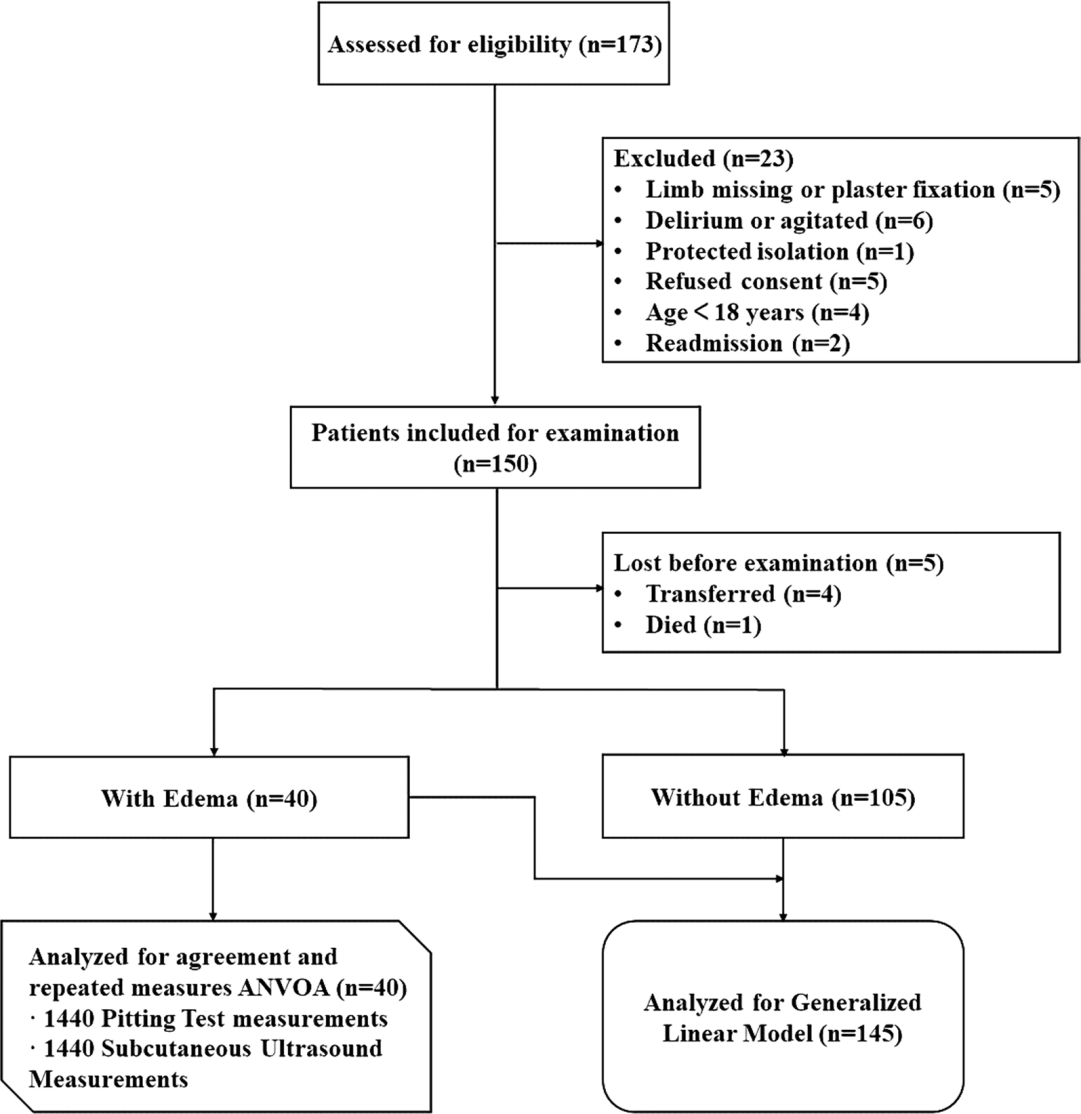
Flowchart showing the study recruitment

Of the 173 patients who were initially recruited, 145 who had both subcutaneous ultrasound and PT examination results were finally enrolled. Figure 2 depicts the flowchart of the patient screening and inclusion. The overall patient population was 60.7% male, with 71.7% aged over 60 years. Regardless of whether the subcutaneous edema was detected at any single site of 36 measure sites by ultrasound, PT, or both methods, patients were assigned to the subcutaneous edema group. Subcutaneous edema was detected by PT followed by US confirmation in 40 patients. Table 1 presents the baseline characteristics and clinical data of the study participants.

**Table 1:** Baseline characteristics and associations with the development of subcutaneous edema

Variables	Total (*n* = 145)	Without Edema (*n* = 105)	Edema (*n* = 40)	Unadjusted	Adjusted
				OR (95%CI)	OR (95%CI)
Sex					
Female	57 (39.3)	45 (42.9)	12 (70.0)	Ref	
Male	88 (60.7)	60 (57.1)	28 (70.0)	1.750 (0.803–3.813)	
Age (years)					
≤ 60	41 (28.3)	30 (28.6)	11 (27.5)	Ref	
> 60	104 (71.7)	75 (71.4)	29 (72.5)	1.055 (0.468–2.378)	
BMI					
< 18	17 (11.7)	12 (11.4)	5 (12.5)	Ref	
18–24	70 (48.3)	54 (51.4)	16 (40.0)	0.711 (0.218–2.321)	
24–28	41 (28.3)	27 (25.7)	14 (35.0)	1.244 (0.365–4.244)	
> 28	17 (11.7)	12 (11.4)	5 (12.5)	1.000 (0.229–4.373)	
Diagnosis					
Respiration failure	38 (26.2)	28 (26.7)	10 (25.0)	2.440 (0.796–7.483)	1.880 (0.435–8.117)
Abdominal infection	5 (3.4)	3 (2.9)	2 (5.0)	4.556 (0.627–33.118)	11.462 (0.517–254.176)
Sepsis	32 (22.1)	11 (10.5)	21 (52.5)	13.045 (4.234–40.191)^*^	24.681 (4.789–127.192)^*^
Cancer surgery	23 (15.9)	22 (21.0)	1 (2.5)	0.311 (0.035–2.746)	0.211 (0.017–2.554)
Others	47 (32.4)	41 (39.0)	6 (15.0)	Ref	Ref
Heart dysfunction					
No	121 (83.4)	93 (88.6)	12 (30.0)	Ref	
Yes	24 (16.6)	12 (11.4)	28 (70.0)	3.321 (1.344–8.209)^*^	
Acute kidney injury					
No	106 (73.1)	86 (81.9)	20 (50.0)	Ref	
Yes	39 (26.9)	19 (18.1)	20 (50.0)	4.526 (2.045–10.017)^*^	
Abdominal infection					
No	129 (89.0)	99 (94.3)	30 (75.0)	Ref	
Yes	16 (11.0)	6 (5.7)	10 (25.0)	5.500 (1.847–16.382)^*^	
Consciousness					
Alert	95 (65.5)	77 (73.3)	18 (45.0)	Ref	
Sedated	39 (26.9)	23 (21.9)	16 (40.0)	2.976 (1.312–6.748)^*^	
Coma	11 (7.6)	5 (4.8)	6 (15.0)	5.133 (1.409–18.704)^*^	
Surgery					
No	33 (22.8)	20 (19.0)	13 (32.5)	Ref	
Yes	112 (77.2)	85 (81.0)	27 (67.5)	0.489 (0.215–1.111)	
Surgery site					
None	34 (23.4)	21 (20.0)	13 (32.5)	Ref	
Abdominal	64 (44.1)	49 (46.7)	15 (37.5)	0.495 (0.201–1.218)	
Thoracic	11 (7.6)	5 (4.8)	6 (15.0)	1.938 (0.491–7.657)	
Skull and Limb	36 (24.8)	30 (28.6)	6 (15.0)	0.323 (0.106–0.987)^*^	
Mechanical ventilation					
No	77 (53.1)	68 (64.8)	9 (22.5)	Ref	
Yes	68 (46.9)	37 (35.2)	28 (77.5)	6.330 (2.724–14.711)^*^	
Vasoactive drugs					
No	109 (75.2)	85 (81.0)	24 (60.0)	Ref	
Yes	36 (24.8)	20 (19.0)	16 (40.0)	2.833 (1.275–6.296)^*^	
APACHE II Score					
≤ 15	62 (42.8)	57 (54.3)	5 (12.5)	Ref	Ref
> 15	83 (57.2)	48 (45.7)	35 (87.5)	8.312 (3.020–22.884)^*^	8.633 (1.922–38.782)^*^
CVP (mmHg)					
≤ 10	44 (30.3)	36 (34.3)	8 (20.0)	Ref	
> 10	29 (20.0)	11 (10.5)	15 (45.0)	0.999 (0.998–1.000)^*^	
Missing	72 (49.7)	58 (55.2)	14 (35.0)		
NT-proBNP (pg/ml)					
≤ 450	62 (42.8)	59 (56.2)	3 (7.5)	Ref	Ref
> 450	83 (57.2)	46 (43.8)	37 (92.5)	15.819 (4.586–54.561)^*^	7.965 (1.755–36.156)^*^
Creatinine (μmol/L)					
≤ 130	117 (80.7)	94 (89.5)	23 (57.5)	Ref	Ref
> 130	28 (19.3)	11 (10.5)	17 (42.5)	6.316 (2.607–15.303)^*^	5.843 (1.401–24.364)^*^
Prealbumin (mg/L)					
> 200	10 (6.9)	9 (8.6)	1 (2.5)	Ref	
150–200	17 (11.7)	16 (15.2)	1 (2.5)	0.563 (0.031–10.117)	
100–150	48 (33.1)	36 (34.3)	12 (30.0)	3.000 (0.344–26.191)	
< 100	70 (48.3)	44 (41.9)	26 (65.0)	5.318 (0.637–44.400)	
Total protein (g/L)					
≥ 60	63 (43.4)	53 (50.5)	10 (25.0)	Ref	
< 60	82 (56.6)	52 (49.5)	30 (75.0)	3.058 (1.358–6.884)^*^	
Albumin (g/L)					
≥ 35	31 (21.4)	29 (27.6)	2 (5.0)	Ref	
< 35	114 (78.6)	76 (72.4)	38 (95.0)	7.250 (1.642–32.004)^*^	

Variables are presented as numbers (percent %)

BMI: Body Mass Index; APACHE II: Acute Physiology, and Chronic Health Evaluation II; CVP: Central Venous Pressure; NT-proBNP: N-terminal pro-B-type natriuretic peptide; OR: Odds Ratio; CI: Confidence Interval

^*^Odds Ratios with significant (*p* < 0.05) associations

### Differences between USEG and PT

**Fig. 3:**
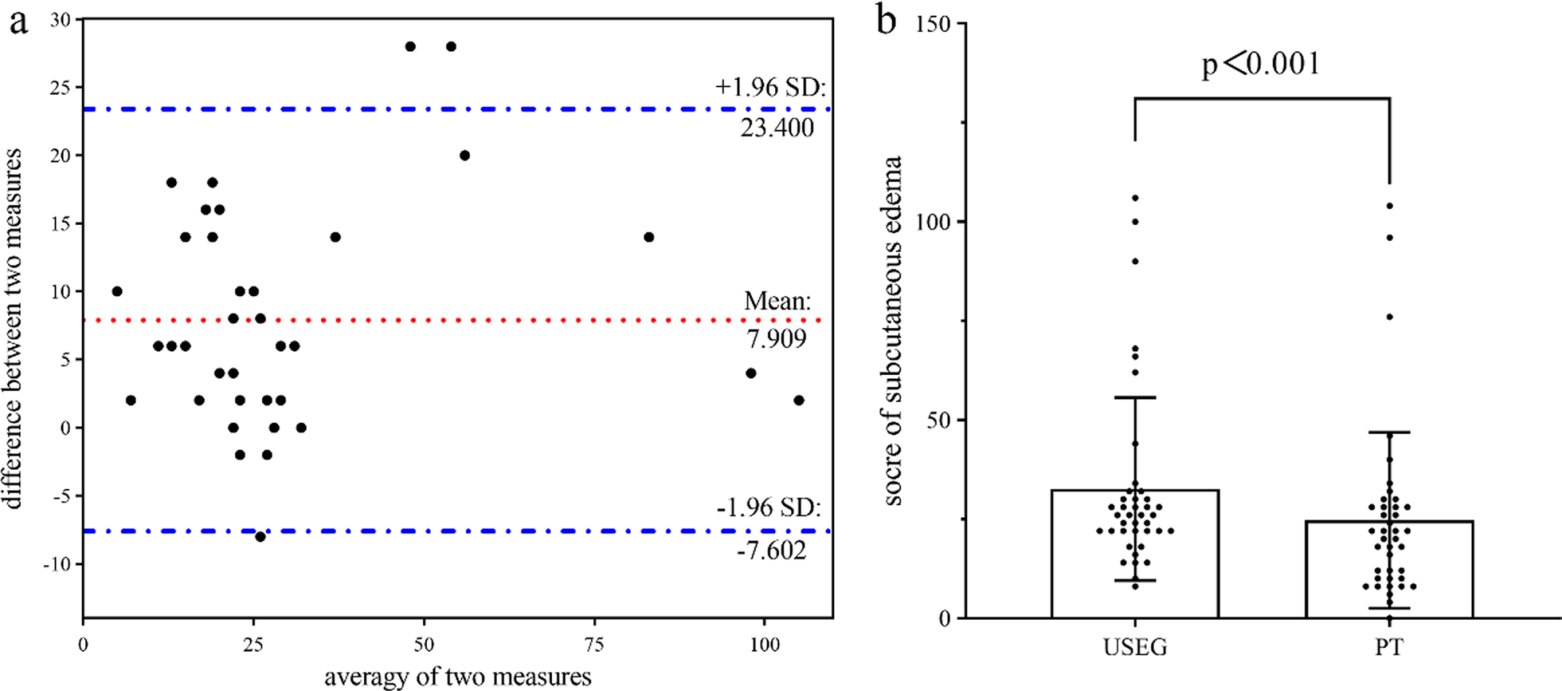
Agreement and differences between USEG and PT. Bland–Altman plot of agreement between USEG and PT (**a**); Paired t-test for subcutaneous edema score between USEG and PT (**b**); USEG: Ultrasonic Subcutaneous Edema Grade; PT: Pitting Test

Although USEG and PT showed excellent agreement (Weighted Kappa = 0.789 [95% CI 0.756–0.823]), in the detection of subcutaneous edema in the 40 patients, USEG detected more instances of edema than PT over 1440 measurements (USEG: 522[36.3%], PT:444[30.8%], *χ*^2^ = 9.477, *p* = 0.002). Specifically, US identified more subcutaneous edema of grade 2 and above (Table 2). In terms of the global edema score, although only 7.5% (3/40) of the points were outside the 95% limits of agreement (Fig. 3a), significant differences between the scores of the two measured were observed (*t* = 6.317, *p* < 0.001) (Fig. 3b). Similarly, significant differences were found between USEG and PT in terms of the measurement of both the whole body and specific body parts, apart from the arms, calves, and feet (Table 3). The whole body showed the highest significant mean difference of 7.900 (95%CI: 5.371–10.429), while the abdominal wall showed the highest significant mean difference of 3.800 (95% CI: 2.547–5.053) among the different body parts.

**Table 2:** Comparison of detection rates between USEG and PT for different edema grades

Subcutaneous Edema (*n* = 1440)	USEG Freq. (%)	PT Freq. (%)	*P*-value	Agreement Weighted Kappa (95% CI)
Grade 0	918 (63.7)	996 (79.2)	< 0.001*	0.789 (0.756, 0.823)
Grade 1	152 (10.6)	172 (11.9)		
Grade 2	112 (7.8)	96 (6.7)		
Grade 3	104 (7.2)	80 (5.5)		
Grade 4	154 (10.7)	96 (6.7)		

USEG: Ultrasonic Subcutaneous Edema Grade; PT: Pitting Test; CI: Confidence Interval

*Chi-square test

**Table 3:** Comparisons of USEG and PT using repeated measures ANOVA

Body part	USEG	PT	*P*-value	Effect size mean difference (95% CI)
Hands	6 (0.8)	4 (0, 8)	0.003	0.700 (0.252, 1.148)
Arms	2 (0, 5.5)	0 (0, 4)	0.108	0.650 (− 0.148, 1.448)
Chest wall	0 (0, 5.5)	0 (0, 2)	0.010	1.050 (0.270, 1.830)
Abdominal wall	10 (6, 14)	6 (2.5, 10)	< 0.001	3.800 (2.547, 5.053)
Thighs	4 (0, 11.5)	2 (0, 8)	0.002	1.300 (0.513, 2.087)
Calves	0 (0, 0)	0 (0, 0)	0.090	0.350 (− 0.057, 0.757)
Feet	0 (0, 5.5)	0 (0, 5.5)	0.711	0.050 (− 0.221, 0.321)
Whole Body	26 (22, 31.5)	21 (10, 28)	< 0.001	7.900 (5.371, 10.429)

USEG: Ultrasonic Subcutaneous Edema Grade; PT: Pitting Test

### Occurrence and distribution of subcutaneous edema

**Fig. 4:**
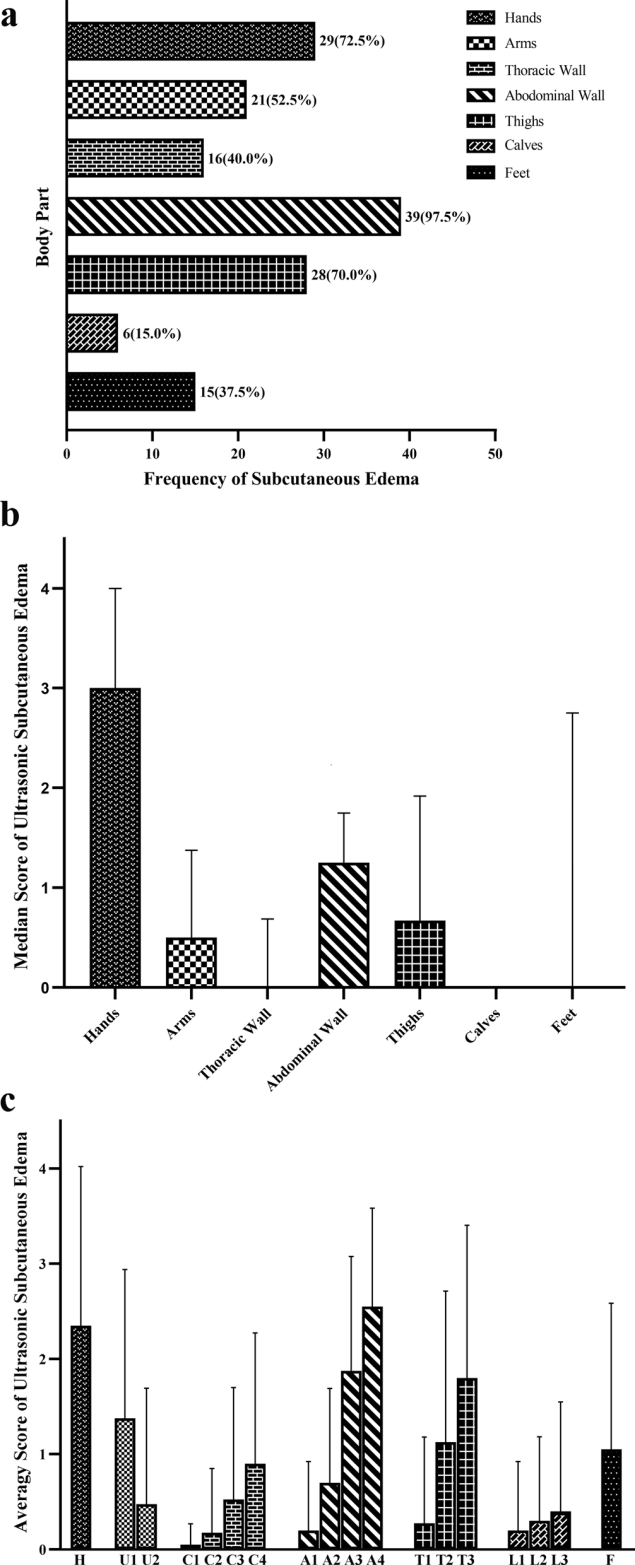
The occurrence and characteristics of subcutaneous edema. Comparison of the frequency of subcutaneous edema among different body parts (**a**); comparison of the ultrasonic subcutaneous edema score among different body parts (**b**); the distribution characteristics of subcutaneous edema in various body parts (**c**)

**Fig. 5:**
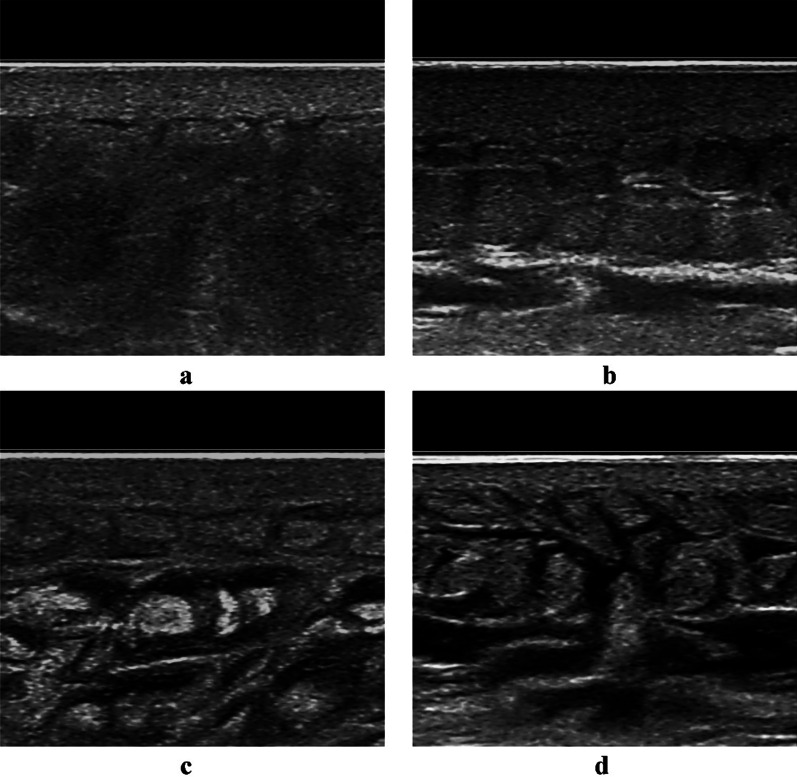
Ultrasonic manifestation of the gravity-dependent features of subcutaneous edema in the abdominal wall. Ultrasonic appearance of subcutaneous edema with Grade 1 in site A1 (**a**); ultrasonic appearance of subcutaneous edema with Grade 2 in site A2 (**b**); Ultrasonic appearance of subcutaneous edema with Grade 3 in site A3 (**c**); ultrasonic appearance of subcutaneous edema with Grade 4 in site A4 (**d**)

Forty out of 145 critically ill patients who were screened were found to have subcutaneous edema on admission to the ICU, representing a 27.6% incidence. Of these 40 patients, 39 had subcutaneous edema of the abdominal wall, an incidence of 97.5%. The proportion of subcutaneous edema in the remaining patients ranged from high to low, including hands (72.5%), thighs (70.0%), arms (52.5%), chest wall (40.0%), feet (37.5%), and calves (15%) (Fig. 4a). In terms of edema severity, the hands had the highest grading score of 3 (0.4), followed by the abdominal wall with 1.25 (0.75, 1.75) and the thigh with 0.67 (0, 1.92) (Fig. 4b). Furthermore, the grading scores increased as the distance to the proximal end of the upper extremities increased, with higher scores also seen in regions closer to the gravity-dependent zones of the thoracic wall, abdominal wall, thighs, and calves (Figs. 4c and 5).

### Relationships between baseline characteristics and development of subcutaneous edema

The results of the generalized linear model analysis for the development of subcutaneous edema are shown in Table 1. Several variables were considered potential risk factors, while only diagnosis (sepsis), APACHE II score (> 15), NT-proBNP (> 450 pg/ml), and creatinine (> 130 μmol/L) were identified as independent risk factors for the development of subcutaneous edema.

### Relationship between the USES and prognosis

**Fig. 6:**
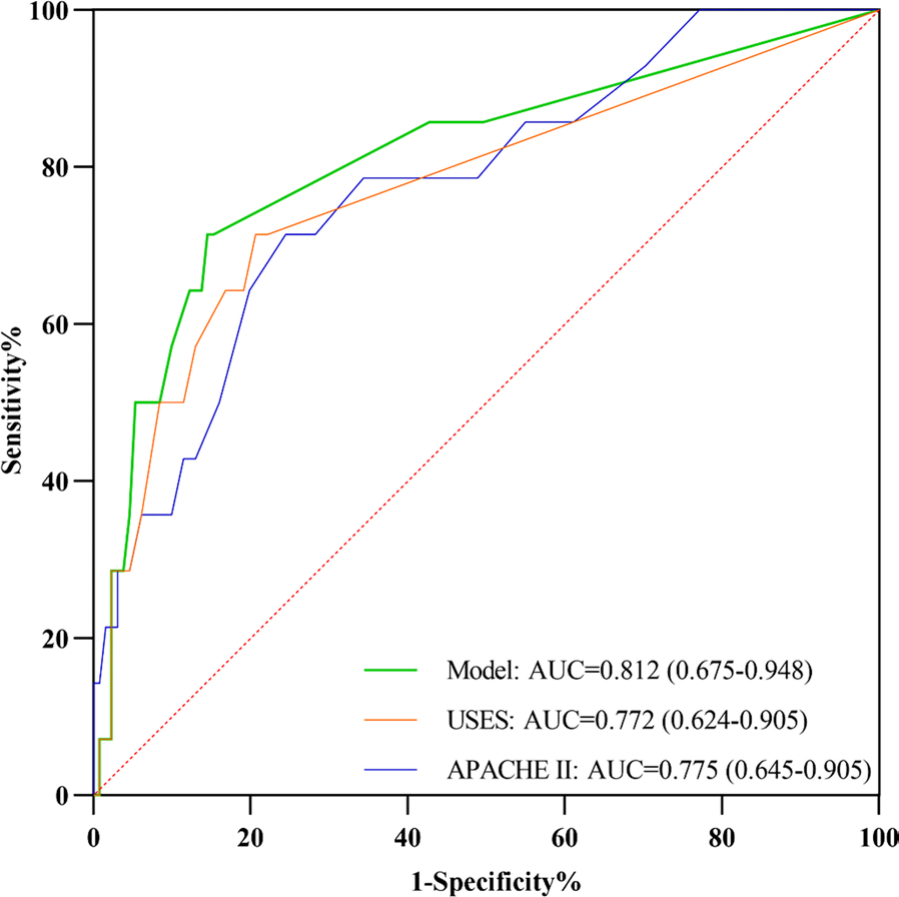
Area under the receiver operating curve for ICU mortality

The association of clinical data with ICU mortality was examined. Besides higher APACHE II score, coma, mechanical ventilation, and vasoactive drugs, increasing subcutaneous edema severity had a 1.038 (95% CI = 1.015–1.062) higher unadjusted risk for ICU mortality, which remained robust in the adjusted analysis (Adjusted OR = 1.028, 95% CI (1.004–1.052) (Table 4). Additionally, the predictive power of the prognostic model constructed with USES and mechanical ventilation (ROC-AUC:0.812, 95%CI [0.675–0.948]) was higher than that of the APACHE II (ROC-AUC:0.775, 95%CI [0.645–0.905]) (Fig. 6).

**Table 4:** Univariable and multivariable analysis for the identification of risk factors for ICU mortality

Variables	Death		Unadjusted	Adjusted
	No(*n* = 131)	Yes(*n* = 14)	OR (95% CI)	OR (95% CI)
USES	0 (0,0)	26.0 (0,36.5)	1.038 (1.015, 1.062)^*^	1.028 (1.004, 1.052)^*^
APACHE II Score	16.0 (13.0, 20.0)	22.5 (18.3,32.8)	5.070 (1.091, 23.555)^*^	–
Coma (Ref: Alert)	7 (5.3)	4 (28.6)	8.476 (1.928, 37.262)^*^	–
Mechanical Ventilation (Yes)	56 (42.7)	12 (85.7)	8.036 (1.729, 37.350)^*^	5.136 (1.029, 25.626)^*^
Vasoactive Drugs (Yes)	29 (22.1)	7 (50.0)	3.517 (1.141, 10.844)^*^	–

Variables were presented as medians and interquartile range [IQR] or numbers (percent %) depending on data distribution

USES: Ultrasonic Subcutaneous Edema Score; APACHE II: Acute Physiology, and Chronic Health Evaluation II; OR: Odds Ratio; CI: Confidence Interval

^*^Odds Ratios with significant (*p* < 0.05) associations

## Discussion

Edema of important organs frequently leads to organ dysfunction, such as pulmonary edema which can cause decreased oxygenation, and cerebral edema which can lead to intracranial hypertension and brain dysfunction, thus triggering earlier clinical attention. Subcutaneous edema, an important component of systemic edema, is common but its clinical importance is frequently overlooked.

There are no standardized criteria for the assessment of patients with subcutaneous edema [7]. The PT is the most common assessment method but has a high inter-rater error in pressure application, and some constant pressure methods require tools that are not appropriate for use on critically ill patients, especially in certain body parts (e.g., the abdominal wall). Because of this, subcutaneous edema assessments are limited to the extremities and lack reliability. Ultrasound records subcutaneous tissue structure and fluid accumulation [12]. Ultrasound can detect subcutaneous edema more accurately and be more patient-specific. We developed a USEG for subcutaneous edema based on the intensity of the tissue echo, the clarity of the tissue structure, and the fluid accumulation characteristics, and compared it to the widely used PT. This showed that the USEG performed better in assessing the onset and severity of edema.

The present study is the first to use a multi-dotted systemic approach to assess subcutaneous edema in critically ill patients, providing a more detailed understanding of fluid distribution and is consistent with holistic treatment. We termed this multi-dotted system "FLUID: Focused Liquid Ultrasonography in Dropsy." The FLUID protocol assesses subcutaneous edema in 7 body parts and 36 sites using USEG. First, FLUID can quantitatively assess the level of subcutaneous edema in a body part. Second, ultrasound assessment of subcutaneous edema is more accurate, faster, and safer than other edema assessment tools and is preferable for dynamic bedside assessment of critically ill patients. USEG scored higher than PT in assessments of the whole body and certain body parts, especially the abdominal wall. The subcutaneous tissue of the abdominal wall is looser than that in other body parts and lacks bony support in the deep subcutaneous region, which affects PT and grading. Ultrasound is not affected by these factors due to its inherent advantages.

Chen et al. [21] found a 24.8% incidence of subcutaneous edema in 1338 ICU patients while Danziger et al. [4] observed an 18% incidence in 12,778 ICU patients. Neither study described how to assess subcutaneous edema or its systemic and local distribution. In this study, 27.6% of critically ill patients had subcutaneous edema on admission. The incidence of subcutaneous edema was highest in the abdominal wall, followed by the hands, thighs, arms, chest wall, and feet. The hands had the most edema, followed by the abdominal wall, thighs, feet, and arms. The abdominal wall and thighs were more prone to subcutaneous edema and higher levels of edema, likely due to the abundance of subcutaneous fat and loose tissue in these areas, especially the abdominal wall, to retain exuded or leaked fluid and form edema when edema-predisposing factors are present.

Distal limb tissues have higher venous pressures and are prone to subcutaneous edema [22, 23]. The findings of the current study revealed various distribution characteristics that may be linked to other primary mechanisms of edema development across multiple disease states, such as increased vascular permeability due to inflammatory reactions, impaired venous return due to cardiac insufficiency, and increased mean filling pressures in the body circulation due to volume overload [8, 24]. These edema mechanisms may affect its distribution. Given the small number of edema patients in this study, further analysis and judgment on the distribution of subcutaneous edema under different pathophysiological conditions are difficult. Larger sample sizes are needed to understand subcutaneous edema in critically ill patients. The higher incidence and severity of edema in the hands compared to the feet may be related to blood pressure measurements and upper limb restraints. The subcutaneous edema of the chest wall, abdominal wall, thigh, and calf was worse when it was closer to the gravity-dependent area, similar to the spatial distribution of increased pulmonary water [25].

The pathophysiological mechanisms underlying edema include decreased plasma oncotic pressure, increased capillary permeability, increased capillary hydrostatic pressure, or lymphatic obstruction [8]. Critically ill patients can have multiple subcutaneous edema risk factors. In this study, APACHE II, NT-proBNP, creatinine, and sepsis were identified as subcutaneous edema risk factors. The more severe the disease, the greater the effects on the circulation, fluid balance, and endocrine function. Critically ill patients tend to experience malnutrition and hypoproteinemia, making them more susceptible to fluid retention and subcutaneous edema [1]. NT-proBNP is a heart failure marker, and its elevation is a risk factor for subcutaneous edema due to volume status, cardiac load, and cardiac function [23]. Acute kidney injury and subcutaneous edema are closely related, and edema severity is correlated with disease severity. Studies have shown that serum creatinine can be used to track subcutaneous edema [26]. In sepsis, the infection can increase endothelial permeability, impair intercellular junctions, and shed polysaccharide-protein complexes, causing capillary leakage, hypervolemia, peripheral edema, and hemodynamic instability [27].

Subcutaneous edema, like organ edema, can induce tissue damage due to oxygen and nutrition exchange, raise the risk of pressure injury in the weight-bearing region, and increase the chance of systemic or local tissue infection, all of which affect the illness outcomes of critically ill patients [4, 21, 28]. Subcutaneous edema intensity was also found to predict prognosis in critically ill patients. The prognostic prediction model was more accurate than the APACHE II score. These results imply that screening and monitoring of subcutaneous edema should be standardized and systematic because critically ill patients may benefit from early detection and intervention of subcutaneous edema.

The present study has some limitations that should be noted. First, the study only assessed subcutaneous edema at the time of ICU admission, without longitudinal observation throughout the ICU stay, therefore lacking a dynamic comprehension of edema status. Second, the study was conducted in a single general care unit with a significant number of surgical patients and a limited sample size, making it difficult to examine subcutaneous edema in varied pathophysiological situations. Third, it was impossible for the ultrasound examiners to be completely blinded to the PT score because the skin manifestations gave the impression of subcutaneous edema. Therefore, different researchers should be assigned for ultrasound image acquisition and interpretation to minimize observer effects in future research. Fourth, the 36 subcutaneous edema measurement sites were not uniformly distributed, with fewer sites in the hands, arms, and feet. Lastly, while the 36 measurement sites are beneficial for understanding subcutaneous edema generally, their use does not favor rapid assessment. Hence, a fast FLUID protocol is needed for clinical application.

## Conclusions

This study describes the FLUID protocol, a semi-quantitative method for measuring and monitoring subcutaneous edema in critically ill patients. ICU patients showed subcutaneous edema in diverse body regions and degrees. Subcutaneous edema was also found to be heterogeneously distributed. Certain body parts, such as the abdominal wall, are gravity-dependent and encourage subcutaneous edema. The APACHE II score, NT-proBNP and creatinine levels, and sepsis were found to be independent risk factors for the development of subcutaneous edema. The severity of subcutaneous edema predicted patient outcomes.

## Data Availability

The data generated from this study will be made available on request to the corresponding authors.

## References

[1] Mangialardi RJ, Martin GS, Bernard GR, Wheeler AP, Christman BW, Dupont WD, Higgins SB, Swindell BB (2000). Hypoproteinemia predicts acute respiratory distress syndrome development, weight gain, and death in patients with sepsis Ibuprofen in Sepsis Study Group. Crit Care Med.

[2] Siddall E, Khatri M, Radhakrishnan J (2017). Capillary leak syndrome: etiologies, pathophysiology, and management. Kidney Int.

[3] Jaffee W, Hodgins S, McGee WT (2018). Tissue edema, fluid balance, and patient outcomes in severe sepsis: an organ systems review. J Intensive Care Med.

[4] Danziger J, Chen K, Cavender S, Lee J, Feng M, Mark RG, Mukamal KJ, Celi LA (2016). Admission peripheral edema, central venous pressure, and survival in critically Ill patients. Ann Am Thorac Soc.

[5] Claure-Del Granado R, Mehta RL (2016). Fluid overload in the ICU: evaluation and management. BMC Nephrol.

[6] Bouchard J, Soroko SB, Chertow GM, Himmelfarb J, Ikizler TA, Paganini EP, Mehta RL (2009). Program to improve care in acute renal disease study G: fluid accumulation, survival and recovery of kidney function in critically ill patients with acute kidney injury. Kidney Int.

[7] Brodovicz KG, McNaughton K, Uemura N, Meininger G, Girman CJ, Yale SH (2009). Reliability and feasibility of methods to quantitatively assess peripheral edema. Clin Med Res.

[8] Koo LW, Reedy S, Smith JK (2010). Patient history key to diagnosing peripheral edema. Nurse Pract.

[9] Wiese J. The patient history: evidence-based approach. Edema In: Tierey L, Henderson M, eds 2005, 1:249–256.

[10] Mayo PH, Copetti R, Feller-Kopman D, Mathis G, Maury E, Mongodi S, Mojoli F, Volpicelli G, Zanobetti M (2019). Thoracic ultrasonography: a narrative review. Intensive Care Med.

[11] Quintavalle PR, Lyder CH, Mertz PJ, Phillips-Jones C, Dyson M (2006). Use of high-resolution, high-frequency diagnostic ultrasound to investigate the pathogenesis of pressure ulcer development. Adv Skin Wound Care.

[12] Lucas VS, Burk RS, Creehan S, Grap MJ (2014). Utility of high-frequency ultrasound: moving beyond the surface to detect changes in skin integrity. Plast Surg Nurs.

[13] Suehiro K, Morikage N, Murakami M, Yamashita O, Samura M, Hamano K (2013). Significance of ultrasound examination of skin and subcutaneous tissue in secondary lower extremity lymphedema. Ann Vasc Dis.

[14] Niimi K, Hirai M, Iwata H, Miyazaki K (2014). Ultrasonographic findings and the clinical results of treatment for lymphedema. Ann Vasc Dis.

[15] Suehiro K, Morikage N, Murakami M, Yamashita O, Ueda K, Samura M, Nakamur K, Hamano K (2014). Subcutaneous tissue ultrasonography in legs with dependent edema and secondary lymphedema. Ann Vasc Dis.

[16] Rastel D, Crebassa V, Rouviere D, Maneglia B (2020). Physician interpretation of ultrasound in the evaluation of ankle edema. Phlebology.

[17] Suehiro K, Morikage N, Yamashita O, Harada T, Samura M, Takeuchi Y, Mizoguchi T, Nakamura K, Hamano K (2017). Correlation between the severity of subcutaneous echo-free space and the amount of extracellular fluid determined by bioelectrical impedance analysis of leg edema. Lymphat Res Biol.

[18] von Elm E, Altman DG, Egger M, Pocock SJ, Gotzsche PC, Vandenbroucke JP, Initiative S (2007). The strengthening the reporting of observational studies in epidemiology (STROBE) statement: guidelines for reporting observational studies. Lancet.

[19] Landis JR, Koch GG (1977). The measurement of observer agreement for categorical data. Biometrics.

[20] Liu H, Wu T (2005). Sample size calculation and power analysis of time-averaged difference. J Mod Appl Stat Methods.

[21] Chen C, Lee J, Johnson AE, Mark RG, Celi LA, Danziger J (2017). Right ventricular function, peripheral edema, and acute kidney injury in critical illness. Kidney Int Rep.

[22] Cho S, Atwood JE (2002). Peripheral edema. Am J Med.

[23] Cooper KL (2011). Care of the lower extremities in patients with acute decompensated heart failure. Crit Care Nurse.

[24] Schroth BE (2005). Evaluation and management of peripheral edema. JAAPA.

[25] Picano E, Scali MC, Ciampi Q, Lichtenstein D (2018). Lung Ultrasound for the Cardiologist. JACC Cardiovasc Imaging.

[26] Chen KP, Cavender S, Lee J, Feng M, Mark RG, Celi LA, Mukamal KJ, Danziger J (2016). Peripheral edema, central venous pressure, and risk of AKI in critical illness. Clin J Am Soc Nephrol.

[27] Wollborn J, Hassenzahl LO, Reker D, Staehle HF, Omlor AM, Baar W, Kaufmann KB, Ulbrich F, Wunder C, Utzolino S (2021). Diagnosing capillary leak in critically ill patients: development of an innovative scoring instrument for non-invasive detection. Ann Intensive Care.

[28] Shah MG, Cho S, Atwood JE, Heidenreich PA (2006). Peripheral edema due to heart disease: diagnosis and outcome. Clin Cardiol.

